# NT-proBNP levels might predict outcomes in severe sepsis, but renal function cannot be ignored

**DOI:** 10.1186/s13054-019-2615-2

**Published:** 2019-11-04

**Authors:** Jiarong Ye, Qianrong Liang, Xiaotu Xi

**Affiliations:** 1 The Second Affiliated Hospital of Guangzhou University of Chinese Medicine and Guangdong Provincial Hospital of Chinese Medicine, No. 111, Dade Road, Yuexiu District, Guangzhou, 510120 China; 2 Guangdong University of Foreign Studies, No. 2, Baiyun Avenue North, Baiyun District, Guangzhou, 510420 China

We read with great interest the article by Carlo Custodero et al. recently published in *Critical Care* [1]. The authors concluded that NT-proBNP levels during the acute phase of sepsis may be a useful indicator of higher risk of long-term impairments in physical function and muscle strength in sepsis survivors. However, the article overlooks the association of NT-proBNP and renal function in septic patients. Studies have shown that acute kidney injury is a common complication of sepsis and is significantly associated with mortality [2, 3], whereas the studies by Gergei et al. [4] and Roberts et al. [5] indicated that NT-proBNP plasma level has shown an exponential increase with declining glomerular filtration rate. Thus, it did not seem persuasive that NT-proBNP could completely predict outcomes without adjusting for the covariate of renal function. We suggest the relationship of the NT-proBNP levels during the acute phase of sepsis and physical function and muscle strength outcomes in sepsis survivors be stratified based on the renal function.

## Data Availability

Not applicable.
